# 

**Published:** 1888-03

**Authors:** 


### ANNOUNCEMENTS.

THE AMERICAN MEDICAL ASSOCIATION.—The next meeting of the Association will be held in Cincinnati, on the first Tuesday in May.

ILLINOIS STATE MEDICAL SOCIETY.—The next annual meeting of the Illinois State Medical Society will occur at Rock Island, on the third Tuesday in May.

THE German Society of Naturalists and Physicians, will hold its next annual meeting, in Cologne, in September, 1888.

AN International Exhibition of Hygiene is to be held in Ostend, Belgium, beginning on June 1, 1888.

THE FOURTH INTERNATIONAL OTOLOGICAL CONGRESS.—The Fourth International Otological Congress will be held in Brussels, Belgium, September 10–16, 1888. The President of the Congress will be Dr. C. Delstauche, and the Secretary, Dr. C. Goris, both of Brussels. It is requested that the titles of papers to be read at the Congress be furnished to the Secretary prior to May 15.

Volume LVI.] MARCH, 1888. [Number 3.

### The CHICAGO MEDICAL JOURNAL AND EXAMINER

EDITOR:
S. J. JONES, M.D., LL.D

COLLABORATORS:

CHICAGO:

PROFESSOR E. ANDREWS, M.D., LL.D.
PROFESSOR J. BARTLETT, M.D.
PROFESSOR W. T. BELFIELD, M.D.
PROFESSOR F. BILLINGS, M.D.
PROFESSOR W. H. BYFORD, A.M., M.D.
PROFESSOR L. CURTIS, A.M., M.D.
PROFESSOR N. S. DAVIS, JR., A.M., M.D.
PROFESSOR O. C. DEWOLF, A.M., M.D.
PROFESSOR E. C. DUDLEY, A.B., M.D.
PROFESSOR C. FENGER, M.D.
PROFESSOR J. N. HYDE, A.M., M.D.
PROFESSOR E. F. INGALS, A.M., M.D.
PROFESSOR F. S. JOHNSON, A.M., M.D.
PROFESSOR H. A. JOHNSON, M.D., LL.D.
PROFESSOR C. W. PURDY, M.D.
PROFESSOR C. G. SMITH, A.M., M.D.
PROFESSOR E. WING, A.M., M.D.

MILWAUKEE, WIS.
PROFESSOR N. SENN, M.D., Ph.D.

WASHINGTON, D. C.:

S. O. RICHEY, M.D.

UNITED STATES ARMY:
SURGEON D. L. HUNTINGTON, M.D.

UNITED STATES NAVY

MEDICAL DIRECTOR J. M. BROWNE, M.D.

MEDICAL DIRECTOR A. L. GIHON, A.M., M.D.

MARINE HOSPITAL SERVICE:
SURGEON S. T. ARMSTRONG, M.D., Ph.D.

